# Interfacial Adipose Tissue in Systemic Sclerosis

**DOI:** 10.1007/s11926-017-0627-y

**Published:** 2017-01-30

**Authors:** Ilja L. Kruglikov

**Affiliations:** Wellcomet GmbH, Karlsruhe, Germany

**Keywords:** Systemic sclerosis, Interfacial white adipose tissue, Myofibroblast, Immature adipocytes, Mature adipocytes, Dermal adipocytes, Adipocyte-myofibroblast transition

## Abstract

**Purpose of Review:**

This review provides a summary of recent insights into the role of the local white adipose tissue (WAT) in systemic sclerosis.

**Recent Findings:**

Adipocytes located in an interfacial WAT area adjacent to fibrotic lesions have an intermediate phenotype and special properties implicated in fibrotic pathology in systemic sclerosis (SSc). The important role of these cells is recognized in different pathologies, such as wound healing, psoriasis, breast cancer, and prostate cancer. Additionally, both immature and mature adipocytes are involved in the appearance of fibroblast-like cells but exhibit different phenotypes and synthetic properties.

**Summary:**

Adipocytes from interfacial WAT adjacent to the fibrotic area in SSc are phenotypically different from bulk adipocytes and are involved in pathogenesis of SSc. Immature and mature adipocytes from this WAT layer differentiate into various types of fibroblast-like cells, making the local ratio of immature to mature adipocytes in interfacial WAT of particular importance in SSc pathogenesis.

## Introduction

Systemic sclerosis (SSc) is an autoimmune rheumatic disease with an incompletely elucidated pathogenesis. Clinical manifestations of SSc include the development of fibrotic lesions in the skin and internal organs [1, 2]. However, the initial events leading to the appearance of myofibroblasts that are responsible for these lesions remain a matter of debate. The main reason for this event is that myofibroblasts can originate from different cells, including fibroblasts, epithelial and endothelial cells, pericytes, and adipocytes [3]. The relative weight of these pathways in different fibrotic lesions in SSc is unknown.

Increasing evidence suggests that white adipose tissue (WAT) is involved in the development of fibrotic lesions in SSc. Such involvement was first hypothesized and investigated at the systemic level. However, serum levels of different adipokines demonstrated no distinct correlations with SSc. Some authors reported no difference between patients with SSc and healthy controls [4], whereas others reported reduced serum adiponectin levels only in diffuse but not in limited SSc [5]. The coefficients of variation for serum levels of adipokines in SSc are generally high, indicating that the investigated groups are heterogeneous, and various additional hidden parameters are potentially primarily responsible for their systemic expression.

Recently, the main interest in this field shifted to the role of adipocytes that are locally adjacent to the lesional tissue. Furthermore, we generally name this adipose tissue “interfacial WAT,” thus differentiating it from the bulk WAT at the same location. In this review, we discuss recent insights in this field.

### Experimental Findings Supporting the Involvement of Interfacial WAT in Fibrosis

The idea that interfacial WAT is involved in fibrosis is supported by multiple experimental findings. First, the appearance of dermal fibrosis is generally connected to some atrophy of the adjacent adipose tissue that generally occurs before the onset of fibrosis. Correlation of these two processes can be observed not only after bleomycin injections [6, 7••] but also after the application of different physical factors, including chronic UV irradiation [8, 9]. This effect is also observed in different knockout models, e.g., in the urokinase-type plasminogen activator receptor (uPAR)-deficient mice: uPAR attenuates adipocyte differentiation, whereas uPAR^−/−^ mice demonstrate significant dermal fibrosis accompanied by a reduction of dermal WAT (dWAT) [6].

Second, immature and mature adipocytes have limited migration capacity in vivo [10]; thus, any direct interaction between adipocytes and other cells appears to be spatially limited to several cell layers. However, very recently, it was reported that adipose stromal cells can be mobilized from WAT through the mechanism of chemotaxis involving different chemokines [11•]. At the same time, these chemokines can be actively secreted by myofibroblasts [12], which not only increases the effective interaction range but also produces a forced flow of immature adipocytes into the fibrotic area. Moreover, various chemokines promote the phenotypic conversion of myofibroblasts and the development of prostatic fibrosis [13].

Third, several recent results demonstrated a correlation between microRNA (miR)-155 and the appearance of fibrotic lesions in SSc. On the one hand, miR-155 is involved in SSc pulmonary fibrosis [14•]. Moreover, miR^−/−^ mice develop significantly milder bleomycin-induced pulmonary fibrosis [14•] and are even resistant to bleomycin-induced skin fibrosis [15•]. On the other hand, high levels of miR-155 suppress adipogenic differentiation and keep preadipocytes in an undifferentiated state [16, 17], thus inhibiting adipogenesis. Interestingly, miR-155 can be stimulated by transforming growth factor β1 (TGF-β1) [17], which is a known fibrotic promoter. Such a mechanism should have a spatially short-range character and thus be connected to adjacent and not systemic WAT.

Fourth, the involvement of adjacent adipose tissue was recently recognized in different pathologies in addition to SSc, including wound healing [18], psoriasis [19], and prostate cancer [20].

Altogether, the adipocytes located near the interface with fibrotic lesions should have some special properties involved in fibrotic pathology. Moreover, significant differences should exist in the role of immature and mature adipocytes in this process.

### Transformation of Adipocytes Into Fibroblast-Like Cells

Fibroblast-like cells appear not only through differentiation of adipose-derived stem cells (ADSCs) but also from mature adipocytes that undergo phenotypic transformation. Replacement of the subcutaneous WAT (sWAT) by connective tissue in SSc was likely first described in [21], where it was also suggested that this process is responsible for the observed skin induration. It was subsequently demonstrated that acute mechanical or thermic injury to adipose tissue causes a quick phenotypic transformation of the mature adipocytes into fibroblast-like cells with a primitive phenotype [22]. Moreover, dedifferentiation of mature adipocytes into fibroblast-like cells surprisingly produced a highly phenotypically homogeneous cell population compared with ADSCs [23•].

Furthermore, the thickness of the superficial WAT layer in SSc murine skin decreases with progression of dermal fibrosis [24, 25]. Induction of SSc in a murine model through bleomycin injections suppresses adipogenesis in ADSCs and simultaneously increases the expression of TGF-β1 [26]. Finally, adipocytes undergo a direct adipocyte-myofibroblast transition, and this transition involves the adiponectin-positive intradermal progenitors from the interfacial WAT layer adjacent to the dermis, i.e., the dermal adipocytes [7••].

This step-by-step process prompted us to conclude that adipocytes located near the dermis/sWAT interface are involved in cutaneous fibrosis.

### Dermal Adipocytes in Cutaneous Fibrosis

Interfacial WAT in dermis is presented by dermal adipocytes, which are the fat cells located at the dermis/sWAT interface where they produce the layered structures in rodents and the typical cone-like structures around the pilosebaceous units that penetrate into the sWAT in humans [27•]. Compared with rodents, the cone-like structure of dWAT in humans provides a significantly increased interface between dermal adipocytes and other components of the dermis.

Skin histology reveals a correlation between the presence of such dermal cones and body areas that are typically susceptible to hypertrophic scarring [28, 29]. Over the past few years, it was shown that dermal adipocytes are involved in different processes, including wound healing, hair follicle cycling, protection against skin infection, homeostatic temperature regulation, appearance of different skin lesions, and skin aging (see recent reviews in [9, 27•, 30]). To highlight their special properties, dermal adipocytes are even grouped into a special dWAT depot [31, 32].

It was assumed that dermal adipocytes are phenotypically different from adipocytes located in the bulk of sWAT [27•]. These cells indeed demonstrate unusually high turnover rates for adipocytes that are comparable with characteristic times of wound healing [18] and hair follicle cycling [33] but not with half-renewal times of 10 years, which are reported for the bulk abdominal adipocytes [34]. Additionally, these cells demonstrate high phenotypic flexibility that may be connected to their intermediate phenotype, which can be compared with an intermediate adipo-epithelial phenotype observed in adipocytes from the mammary gland during and after pregnancy [35]. The existence of such intermediate phenotype corresponds well with the observed dedifferentiation of mature adipocytes into the fibroblast-like “stem” cells exhibiting pronounced multi-lineage potential in vitro [23•].

Local thickness of the dWAT layer is mainly dependent on the number and volume of mature dermal adipocytes. This thickness varies both temporally and spatially, thus making the skin properties only quasi-constant [36]. This thickness is significantly modulated during chronological and photo-induced skin aging [9], follows the hair follicle cycle [36], and demonstrates quick reactions for different physical and pharmacological stimuli [27•]. Different temporal and spatial modifications of dWAT were described in various murine knockout models [9].

### Main Pathways Involved in the Appearance of Myofibroblasts in Dermal Fibrosis

Synthetically active myofibroblasts can appear from resident fibroblasts through fibroblast-myofibroblast transition (FMT) [37], pericytes [38], from epithelial cells through epithelial-mesenchymal transition (EMT) [39], from endothelial cells through endothelial-mesenchymal transition (EndMT) [40], and from some other sources [3]. Most, if not all, of these processes are considered reversible. The reversibility of FMT [41], EMT [42], and EndMT [43] transitions were reported both in vitro and in vivo. Moreover, it was proposed that the same cells can undergo multiple rounds of back and forth transformations, thus demonstrating high phenotypic flexibility [42].

Although atrophy of the adjacent adipose tissue generally precedes the appearance of dermal fibrosis, it is natural to assume that this effect is connected to the production of myofibroblasts from some cells present in adipose tissue. Recently, it was reported that myofibroblasts are produced from adiponectin-positive intradermal progenitors. This process was confirmed by adipocyte phenotype investigations ex vivo and is termed adipocyte-myofibroblast transition (AMT) [7••]. This pathway demonstrates surprisingly rapid dynamics. Twenty-four hours after stimulation with TGF-β1, dermal adipocytes were in a transition state and expressed both perilipin and alpha smooth muscle actin (α-SMA) markers, thus demonstrating an intermediate phenotype. This finding clearly limited the pool of adipocytes involved in myofibroblast appearance to dWAT. Cell fat mapping revealed that the majority of the myofibroblasts accumulated in dermal fibrosis originate from dermal adipocytes [7••]. This information also answers the question about the weighting of different pathways in the local appearance of myofibroblasts in dermal fibrosis. Although these results were obtained in mice, it can be strongly assumed that similar behaviors are noted human dermal lesions in SSc.

Shortly afterwards, it was reported that resistin-like molecule α (RELMα/FIZZ1) triggers dedifferentiation of adipocytes and induces α-SMA expression in these cells [44], suggesting that RELMα/FIZZ1 is involved in AMT [44, 45]. Interestingly, FIZZ1 knockout mice treated with bleomycin exhibit significantly impaired pulmonary fibrosis [46], which is another typical manifestation of SSc. It should be noted that mature adipocytes can be an important source of FIZZ1 in vivo [45], and their appearance in the wound under hypoxic conditions promotes hypertrophic scarring given that this mitogenic factor is generally hypoxia mediated. The same is true for the development of dermal fibrosis in SSc. However, to induce the AMT, the skin environment should be hypoxic immediately before the appearance of dermal fibrosis. Whereas fibrotic skin in SSc indeed demonstrates severe hypoxia [47], the question whether hypoxic skin serves as a necessary condition for AMT should be investigated.

To the best of our knowledge, differentiation of myofibroblasts into adipogenic cells (MAT), which would be the reverse process to AMT, was only reported in vitro to date [48•]. However, such reversibility in vivo can be assumed. As discussed in [27•], AMT is responsible for the disappearance of mammary adipocytes during lactation; in this case, the reappearance of adipocytes after involution of milk-producing lobules is likely connected to MAT.

Recently, it was shown that β-catenin stabilization in fibroblasts from the lower dermis leads to a morphological modification that appears to be typical for the Wnt/β-catenin pathway: reduction of dWAT with simultaneous induction of dermal fibrosis [49, 50•]. β-catenin can be simultaneously activated in different fibroblast lineages performing distinct functions [51]. In this context, transgenic mice expressing Wnt-10b demonstrate typical loss of dWAT preceding the onset of myofibroblast accumulation and dermal fibrosis [52]. Moreover, blockade of the Wnt/β-catenin pathway attenuates bleomycin-induced fibrosis [53]. Although negative regulation of TGF-β1-induced myofibroblasts appearance was reported only for FMT [54], this pathway seems to be of more universal physiological importance and is also potentially involved in AMT.

### Correlation Between dWAT and Dermal Fibrosis

Given that dermal fibrosis is primarily connected to AMT, a correlation between dWAT properties and the severity of dermal fibrosis likely exists. Influence of the mouse strain on the severity of dermal fibrosis in a bleomycin-induced model was investigated in five common strains (B10.A, C3H/HeJ, C57BL/6J, DBA/2, and BALB/c) in 6-week-old female mice [55]. Histological examination revealed considerably more prominent dermal fibrosis in B10.A and C3H/HeJ compared with the other three strains. Later, this study was repeated in female and male BALB/c, C57BL/6, and DBA/2 mice [56]. BALB/c mice produced more severe dermal fibrosis compared with the other two strains, inducing the greatest number of myofibroblasts in the skin. The recruitment of these cells in the C57BL/6 strain was greater than 50% reduced compared with that in BALB/c mice.

Different mouse strains have very different dWAT layers [9] and thus can generally demonstrate different AMT reactions. The dWAT layer is most pronounced in B10.A and C3H/HeJ strains followed by BALB/c; however, it is much thinner in C57BL/6J and DBA/2 mice [55]. This finding can explain the different numbers of recruited myofibroblasts observed in various strains [56]. At the same time, dWAT in mice is a highly dynamic system that is tightly connected to the hair follicle cycle and demonstrates strong thickness variations that are different in various strains and should be assumed to also be gender dependent [9, 57]. Thus, a simple static comparison of dWAT thicknesses in different strains may be insufficient to make a reliable conclusion.

Additional results obtained in [56] revealed sexual dimorphism in bleomycin-induced dermal fibrosis, which was much more pronounced in males than in females. Although the absolute numbers of myofibroblasts accumulated in dermal lesions were strain dependent, myofibroblasts were up to twofold increase in males in every strain. At first glance, this finding appears to be surprising given that the thickness of dWAT demonstrates pronounced sexual dimorphism at least in rodents [58] and is significantly thicker in female mice. It seems natural to assume that the larger reservoir of dermal adipocytes can provide more effective recruitment of myofibroblasts. Although dWAT thickness mainly reflects the number and volume of mature adipocytes, the smaller adiponectin-positive progenitors are mainly involved in AMT [7••]. Gender differences in the ratio immature/mature adipocytes in dWAT are unknown.

These findings demonstrate that dermal adipocytes should play an important role in dermal fibrosis and are potentially responsible for the appearance of myofibroblasts in lesional skin. Moreover, the results obtained in [11•] suppose that immature adipocytes undergo chemoattraction to the lesional area. In addition, the AMT pathway might be of more general pathophysiological importance appearing in different types of fibrosis and thus being one of the initial pathophysiological steps in SSc. Consequently, it can be assumed that this mechanism should appear in other types of fibrosis typical for SSc, including pulmonary fibrosis.

### AMT in Pulmonary Fibrosis

Given that dermal and pulmonary fibroses have similar pathophysiologies and both involve the AMT, it can be assumed that lipofibroblasts play the role of dermal adipocytes in pulmonary fibrosis. These cells are located in the alveolar interstitium, involved in surfactant production [59], and can be recognized by their characteristic lipid droplets. Whereas lung lipofibroblasts are well documented in rodents, their existence in humans is a topic of controversy. Some authors reported the identification of lung lipofibroblasts in humans [60], whereas others failed to confirm these results [61, 62].

Lipofibroblasts have an intermediate adipo-epithelial phenotype [60] similar to that of adipocytes in the mammary gland during pregnancy [35]. This finding should be much more than a simple coincidence given that cells with an adipo-epithelial phenotype in the mammary gland are highly positive for Elf5, which is a master regulator of alveologenesis [35]. Comparing lipofibroblasts with lung myofibroblasts demonstrated that lipofibroblasts have reduced expression of the platelet-derived growth factor receptor-α (PDGFRα) gene, approximately twofold reduced α-SMA expression, and high expression of the G0–G1 switch 2 gene, which is typical for mature adipocytes where it is known to be required for the development of lipid droplets [63]. Additionally, rosiglitazone inhibits α-SMA expression in PDGFRα^+^ lung fibroblasts, suggesting that the activation of PPARγ may promote an adipo-epithelial phenotype typical of lipofibroblasts but not the myofibroblast phenotype [64].

These two phenotypes do not exist separately from each other, and transdifferentiation of lipofibroblasts into myofibroblasts is documented in vitro [65]. This transition is analogous to AMT found in dermal fibrosis and is the key event in bronchopulmonary dysplasia [65]. Such a transition can be induced by different insults, such as hyperoxia, trauma, and infection, and leads to failed alveolarization, suggesting that lipofibroblasts play an important role not only in lung development but also in injury repair.

Thus, transdifferentiation of lipofibroblasts into myofibroblasts in pulmonary fibrosis appears to be a very similar process to AMT originally described in dermal fibrosis. From this point of view, the “adipocyte”-myofibroblast transition is the universal process responsible for the appearance of different types of fibrosis in SSc. In addition, “adipocyte” should be understood as a chimeric cell with some intermediate and variable phenotypes.

### What Can We Learn From the Role of Adipocytes in Cancer Invasion?

Breast cancer demonstrates a characteristic dense collagenous stroma. It is widely accepted that cancer-associated fibroblasts (CAFs) are involved in stroma production [66]. It was hypothesized that peritumoral mature adipocytes adjacent to the front between the malignant and healthy tissue undergo dedifferentiation, producing first the so-called cancer-associated adipocytes (CAAs) and then the fibroblast-like cells from these CAAs [67•]. This transformation occurs through the production of at least one intermediate fibroblast-like phenotype, which was named “adipose-derived fibroblast” (ADF) [67•]. These intermediate cells express fibronectin, collagen I, and the CAF marker FSP-1, but not α-SMA. Thus, these cells are phenotypically different from myofibroblasts [68]. The generation of this phenotype is dependent on activation of the Wnt/β-catenin pathway.

Remarkably, mature adipocytes transform into ADF both in vitro and in vivo, and this transformation is morphologically connected with appearance of small adipocytes and elongated cells containing multiple small droplets. Such morphological features indicated that this intermediate phenotype can be connected with “beiging” of white adipocytes. Under proper conditions, human sWAT can indeed demonstrate the intensive beiging effect. Approximately 50% of omental adipocytes from patients with pheochromocytoma contain multilocular cells expressing UCP1 [69]. Transdifferentiation of white-to-beige adipocytes was also reported in breast cancer [70]. This idea is further supported by a recent finding that depletion of white adipocyte progenitors can induce the beiging effect [71•]. Recently, it was also demonstrated that burn trauma causes progressive beiging of sWAT adipocytes located underneath the burn wound [72].

In contrast, under the influence of tumor-derived factors, ADSCs can differentiate into another CAF subpopulation [73, 74]. These CAFs express a typical α-SMA marker and function in a similar manner to myofibroblasts in wound healing [75]. This fraction is especially important in breast cancer. Enhancement of myofibroblast content in mammary adipose tissue increases its ECM stiffness, whereas such increased stiffness through a positive feedback further promotes myofibroblast differentiation [76]. This process plays a pivotal role in cancer progression [77]. Moreover, adipose stromal cells are the component of the tumor microenvironment that promotes tumor progression, and depletion of these cells inhibits tumor growth [78]. In addition, it is actually not known whether the ADF can further differentiate into typical myofibroblasts.

Although this item was not investigated properly, it can be assumed that similar processes should occur in dermal fibrosis. The appearance of fibrosis should be dependent on the ratio of immature to mature adipocytes in interfacial dWAT, which can be modulated by different internal and external factors. In this context, it should be noted that bleomycin suppresses adipogenesis in ADSCs [26]; thus, this treatment can shift the ratio of immature to mature adipocytes to the immature pool. In addition, CAAs with an intermediate phenotype in breast cancer should be different from intermediate adipocytes expressing α-SMA in dermal fibrosis [7••]. This finding reiterates the chimeric nature of adipocytes, especially in the interfacial area of corresponding WAT [27•].

### Immature and Mature Adipocytes Produce Different Fibroblast-Like Cells

All of these results lead to a general conclusion that adipose-derived progenitors and mature adipocytes should produce different types of fibroblast-like cells. Immature and mature adipocytes exhibit different levels of cytokine expression and drive stem cell signaling in breast cancer cells [79]. Moreover, ADSCs and mature adipocytes differently impact the differentiation status of normal and cancerous breast epithelial cells [80]. In this context, it should be mentioned again that immature adipocytes are subject to chemoattraction to the areas containing myofibroblasts [11•], which can significantly increase their motility. These findings make the ratio of immature to mature adipocytes of particular interest in cancer progression and fibrosis.

It should be noted that this ratio is not constant and is increased in obesity. Moreover, high local expression of TNF-α suppresses adipocyte maturation, increasing the weight of immature adipocytes in the total adipogenic population [79], thus also shifting the weight of different pathways involved in the production of myofibroblasts.

This important item can be illustrated using the recent results obtained for bone morphogenetic protein (BMP), which is the antagonist of TGF-β. BMP signaling is significantly decreased upon bleomycin exposure. In addition, endogenous activation of BMP reduces pulmonary fibrosis [81]. In contrast, BMP4 is induced in human preadipocytes undergoing differentiation [82], regulates the commitment of adipose precursor cells into the white adipocyte lineage, and can even activate the development of the beige phenotype in human precursor cells [83]. Consequently, it can be assumed that BMP should increase the number of differentiating adipocytes, thus reducing the probability that the pathway is connected to the production of myofibroblasts.

Dermal fibroblasts also demonstrate significant spatial phenotypic variations [84], which are connected not only to microenvironment of these cells but also to different origins of their progenitors. Fibroblasts from deeper dermis adjacent to interfacial WAT express more α-SMA markers [85] and thus should contribute more to hypertrophic scarring. These phenotypic differences are gradually dependent on the depth in the dermis, producing a “gradient” of cell phenotypes. In addition, papillary fibroblasts can differentiate into reticular fibroblasts [86], which demonstrates the flexibility of different fibroblast phenotypes. Interfollicular and follicular epidermal cells are also highly heterogeneous. Sharing a common basal-epidermal gene module, these cells simultaneously demonstrate high spatial phenotypic diversity [87].

If immature adipocytes are mainly involved in the production of synthetic active myofibroblasts and the mature adipocytes transform into less synthetically active fibroblast-like cells, the local ratio of immature to mature adipocytes should be more carefully investigated in dermal fibrosis and other SSc pathologies. In this context, more severe bleomycin-induced dermal fibrosis is observed in male compared with female mice [55] and is potentially connected to the higher content of immature adipocytes in males. From this point of view, atrophy of the adipose tissue adjacent to the fibrotic area should be mainly connected to reduction of the number and volume of mature adipocytes and thus should not be a reliable indicator of the severity of dermal fibrosis.

## Conclusions

Local WAT depots are involved in different pathological processes, including psoriasis, different types of cancer, wound healing, and fibrosis. There is increasing evidence that the adipocytes located near the surface of these depots should have phenotypical characteristics and physiological properties that differ from the classical adipogenic phenotype known for bulk sWAT adipocytes. Cells from the interfacial WAT layer adjacent to fibrotic lesions often demonstrate intermediate phenotypes, expressing the marker characteristic both for adipocytes and fibroblasts. This finding is not very surprising—given that these cells should simultaneously interact with classical fibroblasts and classical adipocytes in contrast to the bulk adipocytes.

Although the WAT depots containing these cells differ in dermal and pulmonary fibrosis, the pathophysiological mechanisms of fibrosis in these two diseases appear to be similar. This finding points to the general involvement of adipocytes from interfacial WAT in SSc. Given that the subpopulations of adipocytes in such WAT depots are highly heterogeneous and that the immature and mature adipocytes utilize different pathways in the production of fibroblast-like cells and different migration properties, the structure and content of these adipogenic subpopulations in interfacial WAT depots in SSc should be investigated in more detail in the future experiments.
